# Supplementary material for: Respiration modulates sleep oscillations and memory reactivation in humans
Source: Nat Commun. 2023 Dec 18;14:8351. doi: 10.1038/s41467-023-43450-5 (PMC10728072; doi:10.1038/s41467-023-43450-5)
Supplement: Supplementary file 1 — Supplementary Information [file 41467_2023_43450_MOESM1_ESM.pdf]

Supplementary Information

Respiration shapes sleep oscillations and memory reactivation in humans

Thomas Schreiner[1], Marit Petzka[2,3], Tobias Staudigl[1] & Bernhard P. Staresina[4,5]

1. Department of Psychology, Ludwig-Maximilians-Universität München, Germany
2. Max Planck Institute for Human Development, Germany
3. Institute of Psychology, University of Hamburg, Germany
4. Department of Experimental Psychology, University of Oxford, UK
5. Oxford Centre for Human Brain Activity, Wellcome Centre for Integrative Neuroimaging, Department of Psychiatry, University of Oxford, UK

Correspondence: Thomas.Schreiner@psy.lmu.de

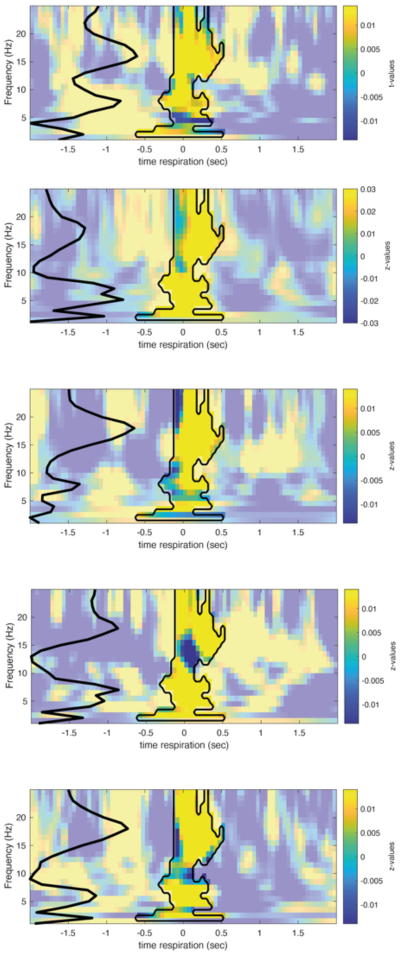

Supplementary Figure 1. Individual time frequency representations of NREM sleep EEG data locked to inhalation peaks for five representative participants. In addition to clear peaks in the SO and spindle range, distinct peaks in the theta range (albeit with varying peak frequencies between 5 and 8 Hz) became apparent (the black line on the left represents z-power across time in the significant cluster indicated by the contour line). The presence of NREM theta bursts in conjunction with enhanced SO power putatively underlies the broad low frequency cluster in Figure 2a. Power enhancement in the spindle range was consistently visible across participants between 13 and 20 Hz, peaking towards higher spindle frequencies (i.e., 17-18 Hz).

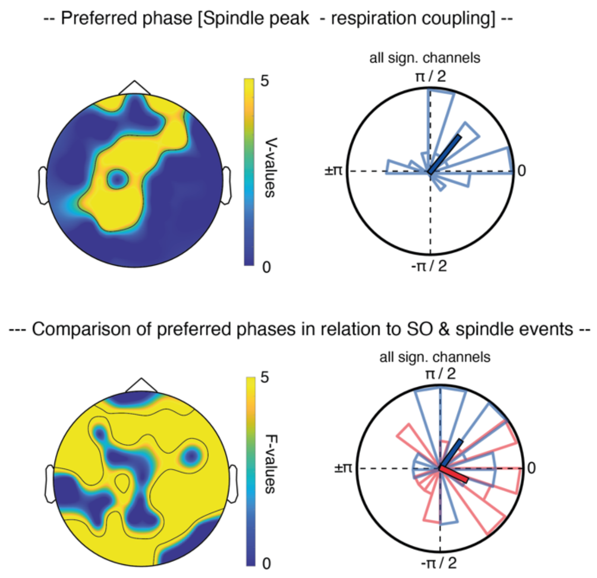

Supplementary Figure 2. Estimating a potential phase shift between SOs and spindles in relation to their modulation by respiration (Top) To assess whether there is a reliable phase shift between SOs and spindles in relation to their modulation by respiration we assessed in a first step the preferred respiration phase for spindle coupling with regards to spindle peaks (the original analysis reported in Fig. 2d with regards to spindle onsets impedes such an analytical procedure due to the proximity of spindle onsets to inhalation peaks and hence SO down-states). Similar to spindle onsets, spindle peaks clustered after the inhalation peak (V-test against 0°, mean V = 5.74 ± 0.25, $p < 0.05$, corrected; mean angle of significant electrodes: 50.45° ± 0.21, mean vector length = 0.57). (Bottom) Next, we assessed using the circular Watson-Williams test whether there would be a phase shift between SOs (i.e., downstates) and spindles (i.e., maximal negative peaks) in relation to their modulation by respiration. We found a significant phase shift across a broad cluster (all $p < 0.05$; corrected) indicating that SOs and spindles clustered at different phases of the respiratory cycle, with SOs emerging before the inhalation peak and spindles following. Source data are provided as a Source Data file.

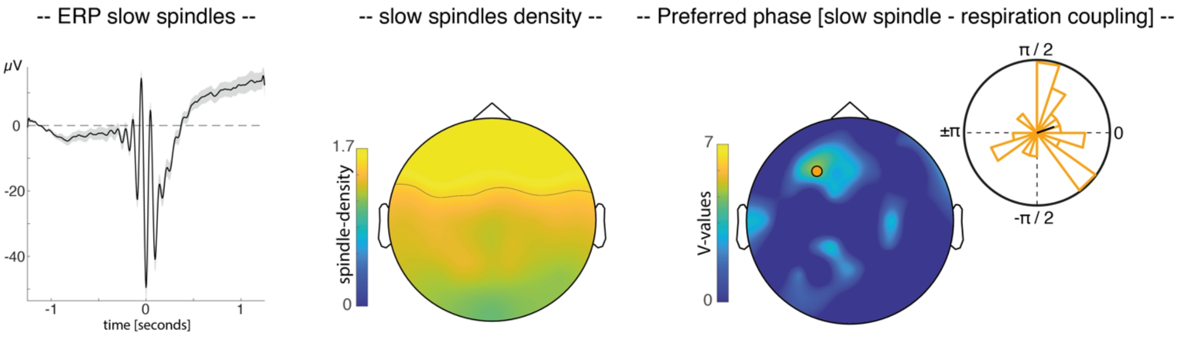

Supplementary Figure 3. Preferred coupling phase for slow spindles. (left) Grand average EEG trace of slow spindles at electrode Fz. (middle) Topographic distribution of slow spindle density [events / minute]. (right) Determining the respiratory phases during the onset of slow spindles across participants revealed no significant non-uniform circular distribution (all $p > 0.05$; displayed in the insert is the electrode with the highest V-value: F3, V-value = 4.7; $p = 0.065$). Source data are provided as a Source Data file.

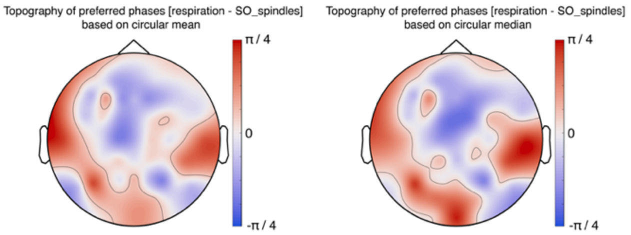

Supplementary Figure 4. Topographies of preferred phases for SO-spindle modulation by respiration (left: based on circular mean; right: based on circular median). The topographies illustrate that SO_spindles tended to occur briefly before the inhalation peak (i.e., 0°) across the cluster were significant phase modulation was detectable (as shown in Fig. 3a).

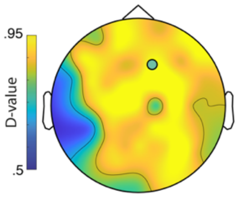

Supplementary Figure 5. Modulation of the likelihood of SOs to group spindles by the respiration phase across electrodes. The number of SO_spindles relative to all SOs at each electrode was binned across the respiration cycle in 20 evenly spaced bins (i.e., from -pi to pi in steps of 0.31 radians). Next, the distributions were averaged per electrode across participants and (non)uniformity of the resulting distribution was assessed using the Kolmogorov-Smirnov test. Non-uniformity peaked at frontal, central and parietal electrodes indicating that the likelihood of SOs to group with spindles was robustly modulated by respiration. Source data are provided as a Source Data file.

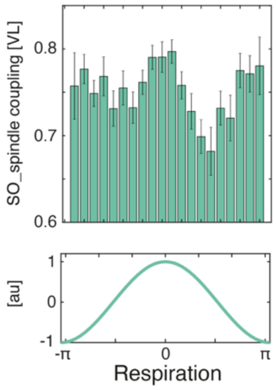

Supplementary Figure 6. The influence of respiration on the consistency of SO-spindle coupling. To assess whether the consistency of SO-spindle coupling is modulated by the respiration phase, we determined in each participant the respiratory peak frequency and filtered the respiratory data (locked to inhalation peaks) around the peak frequency (± 0.05 Hz, two-pass Butterworth bandpass filter, order = three cycles of the low frequency cut-off). Then a Hilbert transform was applied, and the instantaneous phase angle was extracted. Next, we binned the data across the respiration cycle in 20 evenly spaced bins and computed the vector length, hence the amount of SO-spindle phase-amplitude coupling at electrode F2. (Non)uniformity of the resulting distribution was assessed per participant using the Kolmogorov-Smirnov test. We found non-uniform distributions in all participants (all $p < 0.01$; corrected for multiple comparisons across participants using FDR correction), indicating that SO-spindle coupling was moderately impacted by the respiratory phase with the highest vector length around the inhalation peak (i.e., 0°; bars reflect the average vector length ± SEM per bin). It has to be noted that this analysis is based on a modest number of SO_spindles per phase bin, rendering the results preliminary. Future work, making use of whole night recordings, will need to assess their stability on the basis of a higher number of SO_spindle events. Source data are provided as a Source Data file.

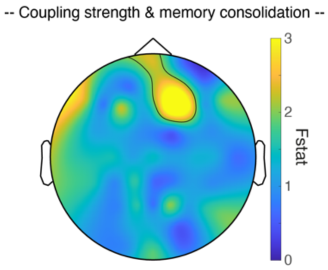

Supplementary Figure 7. Coupling strength and memory consolidation. We conducted, at each electrode, a robust regression across participants between (i) the respiration-SO_spindle coupling strength (i.e., vector length) and (ii) the behavioural levels of associative memory consolidation (i.e., proportion of post-sleep recalled images (out of hits) in relation to pre–sleep memory performance as reported in[13]). While the outcomes of the procedure did not survive correction for multiple comparisons (FDR), it became apparent that the association between respiration-SO_spindle coupling and memory consolidation was strongest at electrode F2 (p = 0.075; F = 3.56), mirroring the relationship between coupling strength and reactivation levels (Fig. 3b). Source data are provided as a Source Data file.

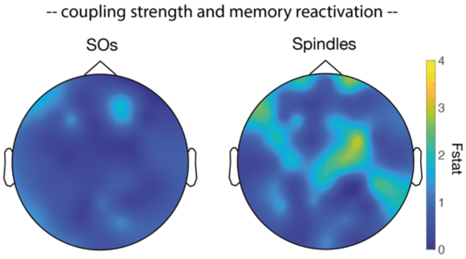

Supplementary Figure 8. Coupling strength [SO-respiration, spindle-respiration] and memory reactivation. Results of a robust regression exhibiting no significant positive relationship between the respiration – SO coupling strength (i.e., vector length) and levels of memory reactivation (all p >0.2) and between respiration – spindle coupling strength and levels of memory reactivation (all p > 0.1). Source data are provided as a Source Data file.

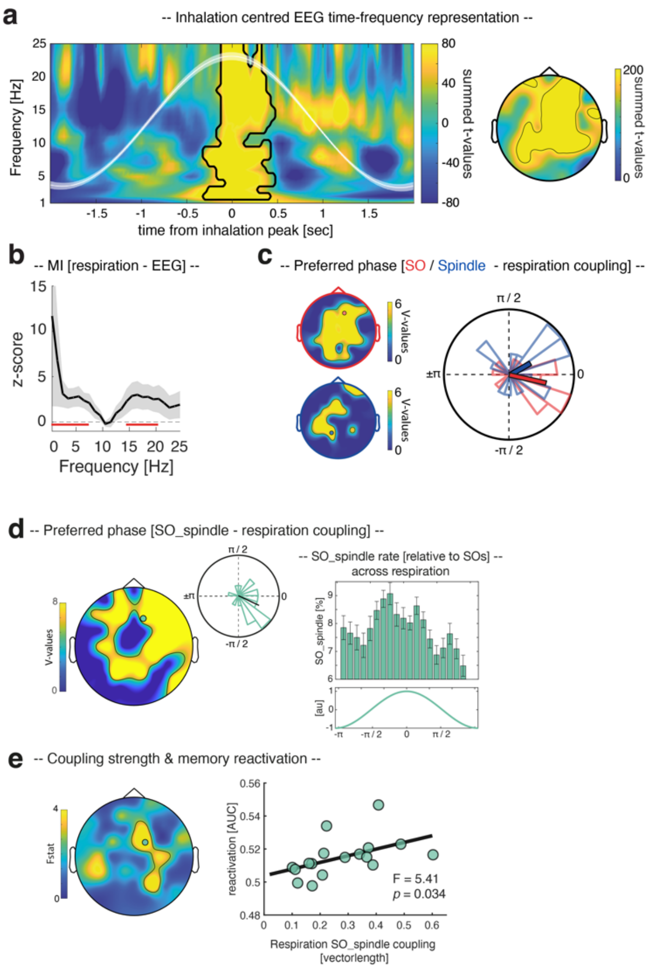

Supplementary Figure 9. Results comprising data from female participants only. (a) TFR of NREM sleep EEG data locked to inhalation peaks, contrasted against random data segments (sum of t-values across all significant electrodes). The contour lines indicate significant clusters ($p < 0.001$, corrected), illustrating enhanced power in the SO_spindle range around the inhalation peak (time = 0). The white waveform depicts the inhalation-peak-locked respiration signal (mean +/- SEM across participants). The topography illustrates the statistical results (summed t-values of the cluster) across electrodes. (b) The modulation index indicates that the phase of respiration influences the EEG amplitude at electrode F2 with local peaks in the SO (0.5 Hz) and spindle range (16 Hz; $z > 1.96$; the red line depicts all significantly modulated frequencies; corrected). (c) Determining the respiratory phases during SO down-states (red topographical insert) and spindle onsets (blue topographical insert) across participants reveals a significant non-uniform circular distribution (SOs and Spindles: $p < 0.05$; corrected using FDR[33]). The example circular plot (electrode F2 for SOs and P1 for spindles, highlighted in the topographies) illustrates the preferred phases for respiration-SO modulation (red: mean angle = -12.61° ± 0.22, mean vector length = 0.56) and respiration-spindle modulation (blue: mean angle = 29.6° ± 0.27, mean vector length = 0.37) in relation to the inhalation peak (i.e., 0°). (d) Determining the respiratory phases during the peak of the detected SO_spindles reveals a significant non-uniform circular distribution ($p < 0.05$; corrected). The example circular plot at electrode F2 illustrates, that the preferred phases of this respiration-SO_spindle modulation peaked right before the inhalation peak (i.e., 0°, mean angle = -19.89° ± 0.21, mean vector length = 0.51). In all participants, SO_spindle rate (relative to SOs) at F2 was non-uniformly distributed across the respiration cycle (all $p < 0.001$; corrected). (e) Results of a robust regression exhibiting a significant positive relationship between the respiration – SO_spindle coupling strength (i.e., vector length) and levels of memory reactivation (i.e., decoding accuracies) at electrode F2 ($F_{1,16} = 5.41$, $p = 0.034$; corrected). Scatter plot illustrates the observed effect. Source data are provided as a Source Data file.

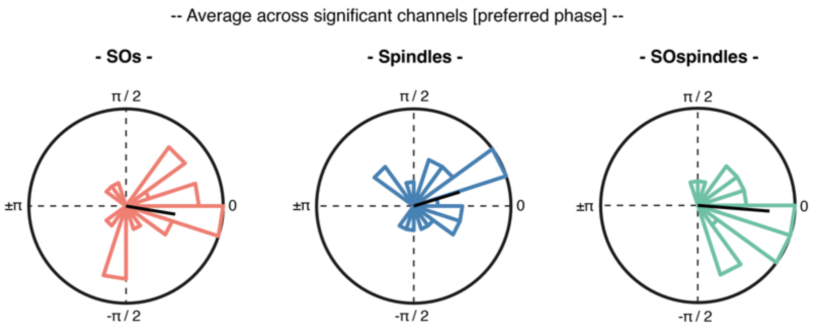

Supplementary Figure 10. Preferred coupling phases across all significant electrodes. Preferred phases of the coupling between respiration and SOs (red, mean angle: -9.4° ± 0.22), spindles (blue, mean angle: 16.95° ± 0.23) and SO_spindle complexes (green, mean angle: -4.5° ± 0.16) averaged across all significant electrodes exhibit similar phase distribution as illustrated in Fig. 2d and Fig. 3a. A significant non-uniform circular distribution was found for SOs, spindles and SO_spindles (V-test against 0°, SOs: V = 10.11, $p < 0.001$; Spindles: V = 9.42, $p = 0.001$; SO_spindles: V = 14.72, $p < 0.001$).

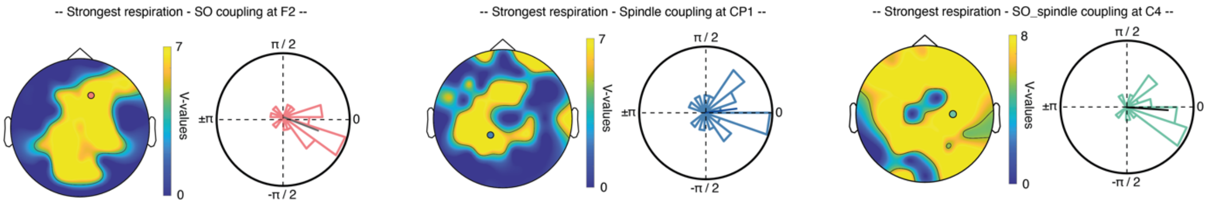

Supplementary Figure 11. Electrodes exhibiting the strongest preferred coupling. The strongest preferred coupling between SOs and respiration (in terms of V-values) became apparent at electrode F2 (red: mean angle: -9.4° ± 0.22; V-test against 0°, V = 10.87, $p < 0.001$). In case of spindles the strongest preferred coupling was located at electrode CP1 (blue: mean angle: 16.95° ± 0.23; V-test against 0°, V = 9.99, $p < 0.001$). For SO_spindles electrode the strongest modulation by respiration was detectable at electrode C4 (green: mean angle: -4.5° ± 0.16; V-test against 0°, V = 13.34, $p < 0.001$)

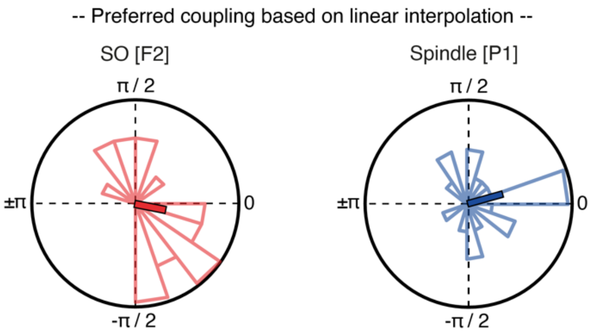

Supplementary Figure 12. Preferred coupling based on double interpolation. To obtain continuous respiration phase angles, peaks (inhalation) and troughs (exhalation) were detected in the normalised respiration time course. Phase angles were linearly interpolated from trough to peak ($-\pi$ to 0) and peak to trough (0 to π) to obtain respiration cycles centred around peak inhalation (i.e., phase 0)[21]. We determined the respiratory phase during detected SO downstates across participants at electrode F2. A significant non-uniform circular distribution became apparent (V = 5.71, p = 0.035), with SO clustering just before the inhalation peak (i.e., 0°; mean angle: -12.06° ± 0.25, mean vector length = 0.29). Next, we assessed the preferred respiration phase for spindle onsets at electrode P1. A significant non-uniform circular distribution was found (V-test against 0°, V = 6.85, p = 0.015), with spindle onsets clustering right after the inhalation peak (mean angle at P1: 14.38° ± 0.25, mean vector length = 0.35). Finally, we directly compared the outcomes with the original Hilbert transformed data (Fig. 2d) using the Watson-Williams test. No significant differences became apparent (SO: F = 0.0013, p = 0.978; Spindles: F = 0.14, p = 0.712).

Sleep stage [%]	MEAN ± SEM
N1	12.5 ± 1.5
N2	43.8 ± 1.6
SWS	21.1 ± 2.6
REM	19.4 ± 2.6
WASO	1.9 ± 0.6
Total Sleep Time [min]	101.6 ± 2.8
# spindles	184.8 ± 12.9
# SOs	459.5 ± 34.5
#SO_spindles	50.1 ± 3.5

Supplementary Table 1. Sleep characteristics (averaged across conditions[13]). Data are means ± SEM. N1, N2: NREM sleep stages N1 & N2, SWS: slow-wave sleep, REM: rapid eye movement sleep, WASO: wake after sleep onset.
