# Supplementary material for: Respiration modulates sleep oscillations and memory reactivation in humans
Source: Nat Commun. 2023 Dec 18;14:8351. doi: 10.1038/s41467-023-43450-5 (PMC10728072; doi:10.1038/s41467-023-43450-5)
Supplement: Supplementary file 3 — Reporting Summary [file 41467_2023_43450_MOESM3_ESM.pdf]

nature portfolio

Corresponding author(s): Thomas Schreiner

Last updated by author(s): Nov 5, 2023

Reporting Summary

Nature Portfolio wishes to improve the reproducibility of the work that we publish. This form provides structure for consistency and transparency in reporting. For further information on Nature Portfolio policies, see our Editorial Policies and the Editorial Policy Checklist.

Statistics

For all statistical analyses, confirm that the following items are present in the figure legend, table legend, main text, or Methods section.

n/a	Confirmed	
☐	☒	The exact sample size (*n*) for each experimental group/condition, given as a discrete number and unit of measurement
☐	☒	A statement on whether measurements were taken from distinct samples or whether the same sample was measured repeatedly
☐	☒	The statistical test(s) used AND whether they are one- or two-sided<br>*Only common tests should be described solely by name; describe more complex techniques in the Methods section.*
☐	☒	A description of all covariates tested
☐	☒	A description of any assumptions or corrections, such as tests of normality and adjustment for multiple comparisons
☐	☒	A full description of the statistical parameters including central tendency (e.g. means) or other basic estimates (e.g. regression coefficient) AND variation (e.g. standard deviation) or associated estimates of uncertainty (e.g. confidence intervals)
☐	☒	For null hypothesis testing, the test statistic (e.g. *F*, *t*, *r*) with confidence intervals, effect sizes, degrees of freedom and *P* value noted<br>*Give P values as exact values whenever suitable.*
☒	☐	For Bayesian analysis, information on the choice of priors and Markov chain Monte Carlo settings
☒	☐	For hierarchical and complex designs, identification of the appropriate level for tests and full reporting of outcomes
☐	☒	Estimates of effect sizes (e.g. Cohen's *d*, Pearson's *r*), indicating how they were calculated

Our web collection on statistics for biologists contains articles on many of the points above.

Software and code

Policy information about availability of computer code

Data collection	MATLAB 2018b ,Mathworks, https://uk.mathworks.com/;<br>Psychophysics Toolbox Version 3;<br>Brain Vision Recorder (Brain Products, Munich, GER);
Data analysis	MATLAB 2018b ,Mathworks, https://uk.mathworks.com/;<br>Fieldtrip Toolbox v.09/01/2020, http://www.fieldtriptoolbox.org/;<br>CircStat Toolbox v.1, https://www.jstatsoft.org/article/view/v031i10;<br>BreathMetrics: a respiratory signal processing toolbox; https://github.com/zelanolab/breathmetrics<br>MVPA Light; https://github.com/treder/MVPA-Light<br><br>All custom code to reproduce the central findings of this study are available at the Open Science Framework (https://doi.org/10.17605/OSF.IO/D6YHB)102.

For manuscripts utilizing custom algorithms or software that are central to the research but not yet described in published literature, software must be made available to editors and reviewers. We strongly encourage code deposition in a community repository (e.g. GitHub). See the Nature Portfolio guidelines for submitting code & software for further information.

Data

Policy information about availability of data

All manuscripts must include a data availability statement. This statement should provide the following information, where applicable:

- Accession codes, unique identifiers, or web links for publicly available datasets
- A description of any restrictions on data availability
- For clinical datasets or third party data, please ensure that the statement adheres to our policy

This study comprises the re-analysis of an existing dataset (Schreiner et., al 2021). All processed data supporting the findings of this study are publicly available at the Open Science Framework (https://doi.org/10.17605/OSF.IO/D6YHB). While publicly sharing the raw data is prohibited due to ethics protocols , data may be shared upon request. To obtain the data, please contact the corresponding author, Thomas Schreiner (Thomas.Schreiner@psy.lmu.de). Source data are provided with this paper.

Research involving human participants, their data, or biological material

Policy information about studies with human participants or human data. See also policy information about sex, gender (identity/presentation), and sexual orientation and race, ethnicity and racism.

Reporting on sex and gender	20 participants (17 female, 3 male) took part in the experiment. No hypotheses regarding the effects of sex or gender were included in the study. Restricting the sample to female only participants did not alter the results (Supplementary Fig. 9) . Information regarding participants' gender were acquired by questionnaires.
Reporting on race, ethnicity, or other socially relevant groupings	No socially constructed or socially relevant categorization variables were used in this study.
Population characteristics	All participant demographic information was added to the Methods section. In brief, twenty healthy, right-handed participants (mean age: 20.75 ± 0.35 ; 17 female) took part in the experiment.
Recruitment	Participants were recruited online via University of Birminghams' SONA system (https://www.sona-systems.com). All individuals who expressed interest in participating in the study were screened and those who met the inclusion criteria were recruited. Potential sources of bias could be present in that individuals who were interested in research and / or had sufficient time may be more likely to self-select and volunteer for the study.
Ethics oversight	The study was approved by the University of Birmingham Research Ethics Committee.

Note that full information on the approval of the study protocol must also be provided in the manuscript.

Field-specific reporting

Please select the one below that is the best fit for your research. If you are not sure, read the appropriate sections before making your selection.

☒ Life sciences ☐ Behavioural & social sciences ☐ Ecological, evolutionary & environmental sciences

For a reference copy of the document with all sections, see nature.com/documents/nr-reporting-summary-flat.pdf

Life sciences study design

All studies must disclose on these points even when the disclosure is negative.

Sample size	Sample size was set to 20 participants based upon previous sleep and memory studies (Ngo et al, 2015; Helfrich et al, 2018).
Data exclusions	Five participants had to be excluded due to insufficient sleep (less than 30 minutes sleep during one of the sessions).
Replication	No independent replication was undertaken. However, findings replicated across multiple subjects, and findings also replicated from multiple sessions within some subjects.
Randomization	Across all participants the order of experimental sessions was randomized (counterbalanced). Statistical controls were used when applicable. Replication mainly comprised determining whether the reported effects were present across participants.
Blinding	The experimenters were not blinded to session allocation (i.e., order of session per participant). This was not relevant to the study, as there were no differences in outcomes expected (i.e., differences in memory performance or electrophysiological measures across sessions) , safely excluding any potential bias.

Reporting for specific materials, systems and methods

We require information from authors about some types of materials, experimental systems and methods used in many studies. Here, indicate whether each material, system or method listed is relevant to your study. If you are not sure if a list item applies to your research, read the appropriate section before selecting a response.

Materials & experimental systems

n/a	Involved in the study
☒	☐ Antibodies
☒	☐ Eukaryotic cell lines
☒	☐ Palaeontology and archaeology
☒	☐ Animals and other organisms
☒	☐ Clinical data
☒	☐ Dual use research of concern
☒	☐ Plants

Methods

n/a	Involved in the study
☒	☐ ChIP-seq
☒	☐ Flow cytometry
☒	☐ MRI-based neuroimaging
